# Betaine Inhibits Interleukin-1β Production and Release: Potential Mechanisms

**DOI:** 10.3389/fimmu.2018.02670

**Published:** 2018-11-20

**Authors:** Yaoyao Xia, Shuai Chen, Guoqiang Zhu, Ruilin Huang, Yulong Yin, Wenkai Ren

**Affiliations:** ^1^Guangdong Provincial Key Laboratory of Animal Nutrition Control, Institute of Subtropical Animal Nutrition and Feed, College of Animal Science, South China Agricultural University, Guangzhou, China; ^2^University of Chinese Academy of Sciences, Beijing, China; ^3^Laboratory of Animal Nutrition and Health and Key Laboratory of Agro-Ecology, Institute of Subtropical Agriculture, Chinese Academy of Sciences, Changsha, China; ^4^Jiangsu Co-Innovation Center for Important Animal Infectious Diseases and Zoo Noses, Joint International Research Laboratory of Agriculture and Agri-Product Safety of Ministry of Education of China, College of Veterinary Medicine, Yangzhou University, Yangzhou, China; ^5^Academics Working Station at The First Affiliated Hospital, Changsha Medical University, Changsha, China

**Keywords:** betaine, caspase-8, IL-1β, inflammation, inflammasome

## Abstract

Betaine is a critical nutrient for mammal health, and has been found to alleviate inflammation by lowering interleukin (IL)-1β secretion; however, the underlying mechanisms by which betaine inhibits IL-1β secretion remain to be uncovered. In this review, we summarize the current understanding about the mechanisms of betaine in IL-1β production and release. For IL-1β production, betaine affects canonical and non-canonical inflammasome-mediated processing of IL-1β through signaling pathways, such as NF-κB, NLRP3 and caspase-8/11. For IL-1β release, betaine inhibits IL-1β release through blocking the exocytosis of IL-1β-containing secretory lysosomes, reducing the shedding of IL-1β-containing plasma membrane microvesicles, suppressing the exocytosis of IL-1β-containing exosomes, and attenuating the passive efflux of IL-1β across hyperpermeable plasma membrane during pyroptotic cell death, which are associated with ERK1/2/PLA_2_ and caspase-8/A-SMase signaling pathways. Collectively, this review highlights the anti-inflammatory property of betaine by inhibiting the production and release of IL-1β, and indicates the potential application of betaine supplementation as an adjuvant therapy in various inflammatory diseases associating with IL-1β secretion.

## Introduction

Immune-cell-mediated inflammation is essential for host protection against infections and injuries. The immune system will coordinate the unanimous reaction to eliminate pathogens and restore tissue integrity in response to infections. Innate immune cells (e.g., macrophages) form the first line of defense to identify initial infections and injuries, and then to promote the recruitment of additional immune cells (e.g., T cells) by releasing cytokines and chemokines. The interleukin (IL)-1 family cytokines [for the history of IL-1, refer to the review (1)] are the central mediators of inflammation and play crucial roles in aforementioned processes (2, 3). Notably, IL-1β is the best-characterized and most extensively studied pro-inflammatory cytokine in IL-1 family, and plays a vital role in host defense in response to infections and injuries (4, 5). IL-1β is mainly produced by the activated-inflammatory cells (e.g., monocytes, microglia, and macrophages) with a multistep process involving synthesis of immature pro-IL-1β, proteolytic cleavage to mature IL-1β, and finally release into the extracellular environment (1, 6).

Betaine (trimethylglycine) is a stable and nontoxic natural compound (7) and shows a wide distribution within phylogenetically distant organisms from microorganisms to animals (8). Betaine is the basic biochemical molecule of the methionine/homocysteine cycle (9), and serves as a methyl group donor in transmethylation [a process catalyzed by betaine-homocysteine methyl transferase (BHMT)], and is essential for choline-mediated one-carbon metabolism, cell membrane integrity, signal transduction and neurotransmitter synthesis (10, 11). Besides, betaine is an important osmoprotectant, which modulates cell volume, and protects cells, proteins and enzymes from osmotic/ionic stress (12, 13). Notably, betaine has been proven to be effective against many inflammatory diseases (e.g., diabetes and NAFLD) with its anti-inflammatory functions (14, 15). Interestingly, betaine participates in alleviation of inflammation by lowering secretion of pro-inflammatory cytokines (e.g., IL-1β, TNF-α, IL-6, and IL-23) (16). Increasing studies have reported that betaine dampens activity of nuclear factor kappa B (NF-κB) to block the expression of genes involved in inflammation, such as IL-1β, COX-2, and iNOS (17, 18). Additionally, betaine can restore normal energy metabolism to relieve systemic low-grade inflammation (e.g., obesity and diabetes) (15, 19–22); and for the main metabolic pathways and crucial mediators modulated by betaine in chronic inflammation, refer to the review (23). Since IL-1β is the central mediators of inflammation, it is worthy of lowering IL-1β secretion to alleviate inflammation. Although, betaine inhibits NOD-like receptor (NLRP) 3 inflammasome activation, which highly shapes the pro-IL-β maturation (24–26), the underlying mechanisms by which betaine inhibits IL-1β secretion are still not fully understood.

In this review, we discuss the potential mechanisms by which betaine could inhibit the IL-1β production through canonical and non-canonical inflammasome-mediated processing of IL-1β, and inflammasome-independent sources of IL-1β. Then, we highlight the evidence about the key roles of betaine in inhibition of IL-1β release with special emphasis on the involved mechanisms, including exocytosis of IL-1β-containing secretory lysosomes, shedding of IL-1β-containing plasma membrane microvesicles, exocytosis of IL-1β-containing exosomes, and passive efflux of IL-1β across hyperpermeable plasma membrane during pyroptotic cell death.

## Betaine inhibits IL-1β production

IL-1β production involves the synthesis of immature pro-IL-1β (31 kD) by the recognition of toll-like receptors (TLRs) (27), and proteolytic cleavage to mature IL-1β (17 kD) by caspase-1 (28). In this section, we summarize the mechanisms by which betaine inhibits IL-1β production, including the canonical and non-canonical inflammasome-mediated processing of IL-1β and inflammasome-independent sources of IL-1β.

### Betaine in canonical inflammasome-mediated processing of IL-1β

As we discussed in the previous part, unprovoked immune cells (when they are under steady-state conditions), like monocytes and macrophages, do not express or just express extremely low level of IL-1β. However, the pro-inflammatory triggers [e.g., (tumor necrosis factor) TNF, IL-1α/6, and TLR-ligands] promote the activation of NF-κB, and expression of IL-1β (29, 30).

**Figure 1:**
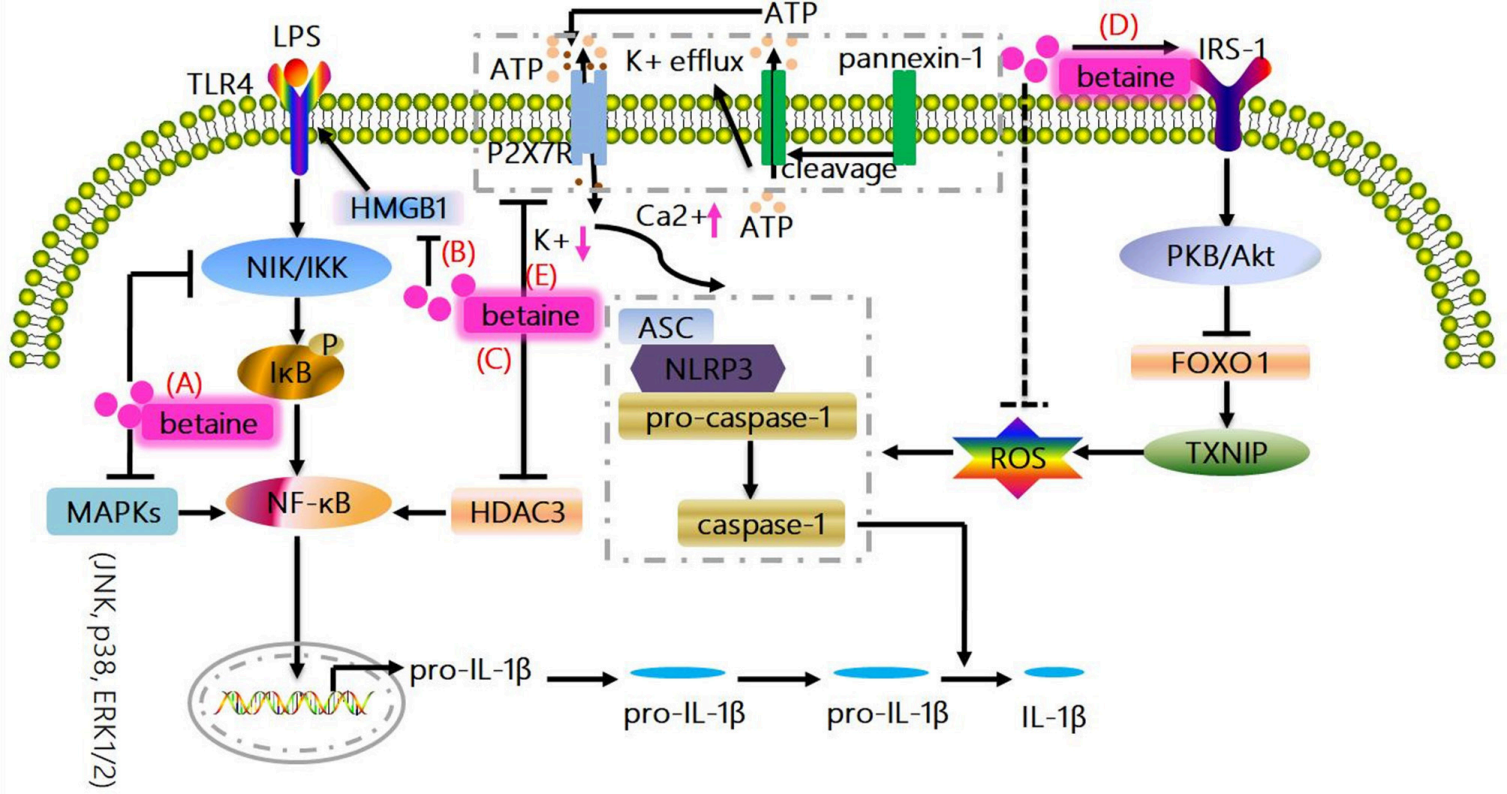
Canonical mechanisms whereby betaine inhibits IL-1β production. **(A)** MAPKs (JNK, p38, and ERK1/2) are responsible for the expressions of pro-inflammatory cytokines; and NIK/IKK relieves the inhibition of IκB, resulting in the activation of NF-κB to promote the up-regulation of IL-1β. Betaine suppresses NF-κB activity and IL-1β expression *via* inhibiting MAPKs and NIK/IKK. **(B)** Betaine inhibits the mRNA and protein expression of HMGB1 which regulates the activation of TLR4, which are involved in NF-κB activation. **(C)** Betaine suppresses HDAC3 which binds to IκBα to activate NF-κB. **(D)** NLR-driven ASC recruitment drives pro-caspase-1 activation leading to pro-caspase-1 cleavage and caspase-1 maturation, subsequently caspase-1 cleaves pro-IL-1β to produce the bioactive IL-1β. Betaine enhances IRS-1 phosphorylation to activate PKB/Akt which results in FOXO1 inactivation leading to a FOXO1 inhibition of TXNIP, which functions as the endogenous inhibitor of ROS-scavenging protein, thereby inhibiting the activation of NLRP3 inflammasome. **(E)** Moreover, the activation of NLRP3 inflammasome is related to the K^+^ efflux caused by ATP-mediated P2X7R activation. Betaine suppresses NLRP3 activation through maintaining cytosolic normal K^+^ levels. JNK, c-Jun NH2-terminal kinase; p38, protein 38; ERK1/2, extracelluar signal-regulated kinase; IL-1β, interleukin-1β; NF-κB, nuclear factor kappa B; MAPKs, mitogen-activated protein kinases; NIK/IKK, nuclear factor-including kinase/IκB kinase; HMGB1, high-mobility group box 1; TLR, toll-like receptor; HDAC3, histone deacetylases 3; IRS-1, insulin receptor substrate 1; FOXO1, forkhead box O 1; TXNIP, thioredoxin interacting protein; ROS, reactive oxygen species; P2X7R purinergic; ligand-gated ion channel 7 receptor; NLRP3, NOD-like receptor.

Betaine suppresses NF-κB activity and its downstream genes (e.g., *IL-1*β) expression *via* inhibiting mitogen-activated protein kinases (MAPKs) and nuclear factor-including kinase/IκB kinase (NIK/IKK) in the aged rats and rat endothelial YPEN-1 cells (18, 31) (Figure 1A). MAPKs include c-Jun NH2-terminal kinase (JNK), protein 38 (p38) and extracelluar signal-regulated kinase (ERK1/2), and are responsible for the expressions of pro-inflammatory cytokines (32); and NIK/IKK relieves the inhibition of IκB, leading to the activation of NF-κB (33). Moreover, betaine also inhibits TLRs which are involved in NF-κB activation (Figure 1B). For instance, in LPS (a TLR4 ligand)-stimulated RAW264.7 cells, betaine suppresses the activation of NF-κB (34). Mechanistically, in high-fat-diet-induced NAFLD rat models, betaine inhibits the mRNA and protein expression of high-mobility group box 1(HMGB1) in liver tissues, which regulates the activation of TLR4 (35) (Figure 1B). Additionally, in fructose-fed rat astrocytes, it is supposed that betaine could suppress the expression of histone deacetylases 3 (HDAC3), which binds to IκBα to activate NF-κB (24) (Figure 1C). Collectively, *IL-1*β is one of the most important downstream genes of NF-κB; and increasing *in vitro* and *in vivo* studies have demonstrated that betaine dampens NF-κB activation. Thus, these findings indicate that betaine inhibits IL-1β production *via* inhibition of NF-κB signaling pathway.

The activated caspase-1 in the canonical inflammasome complex is the most extensively identified mechanism for IL-1β processing. In details, the canonical inflammasomes contain cytosolic sensor molecules [NOD-like receptor (NLR) and absent in melanoma (AIM) 2-like receptor (ALR) families], caspase-1, and adaptor molecule ASC (36, 37). Mechanistically, NLR-driven ASC recruitment drives pro-caspase-1 activation resulting in pro-caspase-1 cleavage and caspase-1 maturation. Then caspase-1 cleaves pro-IL-1β to produce the mature forms of IL-1β (38–40). NLRs include NLRP1, NLRP3 (the main platform for IL-1β processing), NLRP6, NLRP7, NLRP12, and NLRC4, which are all suggested to coordinate inflammasome signaling and induce IL-1β production under specific conditions (41–44), though there exist negative effects (45–48). Indeed, the recognition of pathogen- and/or danger-associated molecular patterns (PAMPs and/or DAMPs) (e.g., bacterial toxins, fungal products, ATP, silica, ceramide, cholesterol crystals, and amyloid β) provokes the inflammasome-mediated IL-1β production, especially *via* NLRP3 activation (49–51). NLRP3 has a specific disulfide bond between Cys-8 and Cys-108 that may involve in modulation of NLRP3 activation by reactive oxygen species (ROS) based on a high resolution structure analysis (52). Likewise, a study showed that liposomes could induce NLRP3 inflammasome activation by mtROS (53). Extensive studies reveal that the activation of NLRP3 inflammasome is associated with the decreased cytosolic K^+^ level (called K^+^ efflux) caused by ATP-mediated purinergic ligand-gated ion channel 7 receptor (P2X7R) activation (39, 54–56). K^+^ efflux also regulates NLRC4 and NLRP1b activation (57, 58); however, how K^+^ concentration regulates the assembly of NLRP3 into functional inflammation is unclear. Thus, it is critical to impede IL-1β processing by suppressing the activation of NLRP3 inflammasome.

Increasing studies prove that betaine blocks NLRP3 inflammasome activation *in vivo* (24–26, 59, 60). Betaine suppresses NLRP3 inflammasome involving a forkhead box O 1(FOXO1) inhibition of thioredoxin interacting protein (TXNIP) which functions as the endogenous inhibitor of ROS-scavenging protein, enhancing ROS to induce NLRP3 inflammasome assembly in macrophages from insulin-resistant obese db/db mice (15, 61). Mechanistically, we suggest that betaine enhances insulin receptor substrate 1 (IRS-1) phosphorylation to indirectly activate PKB/Akt, which results in FOXO1 inactivation through phosphorylating the activated FOXO1 to induce its transfer from the nucleus into the cytoplasm, leading to the inhibition of NLRP3 inflammasome (19, 26) (Figure 1D). Additionally, emerging evidences have demonstrated that betaine enhances/restores Na^+^-K^+^-ATPase activity which maintains low Na^+^ and high K^+^ cell homeostasis (62, 63), and similarly, reduces K^+^ efflux (64); therefore, we speculate that betaine suppresses NLRP3 activation through maintaining cytosolic normal K^+^ levels (Figure 1E). Hence, betaine inhibits IL-1β processing *via* blocking NLRP3 inflammasome activation directly or through IRS-1/PKB/Akt/FOXO1 signaling pathway to resist the activation of NLRP3 indirectly.

Intriguingly, in macrophages, enhanced pro-IL-1β processing is associated with caspase-1; however, caspase-1-independent mechanism of IL-1β processing accounts for IL-1β secretion in neutrophils (65). Mechanistically, in neutrophils, IKKβ-driven NF-κB positively modulates pro-IL-1β mRNA and serine protease inhibitor genes transcription whose products block the proteinase (PR3) activity, which can process pro-IL-1β (65, 66). Unfortunately, whether betaine affects serine protease inhibitor genes remains largely unexplored. Other mechanisms that beyond the core machinery of the inflammasome complex, are also associated with inflammasome assembly, including NLRP3. For example, double-stranded RNA-dependent protein kinase (PKR) and guanylate binding protein (GBP) 5 both contribute to the NLRP3 oligomerization and activation *via* physically interacting with certain inflammasome components (67, 68). However, whether betaine inhibits NLRP3 inflammasome activation through influencing their physical interaction with several components (e.g., ASC) still require comprehensive investigation. Various cell types and stimuli determine the activation of NLRP3 inflammasome. For example, the NLRP3 inflammasome can be spontaneously activated by primary stimulation of human monocytes, during which PAMP and DAMP provide ample signals to produce bioactive IL-1β (69). *M. tuberculosis* still triggers the maturation and production of IL-1β in human monocyte-derived macrophages (70), although it inhibits inflammasome activation (71); however, murine microglia primed with conditioned media from cultures of macrophages infected with *M. tuberculosis* result in caspase-1 activation and IL-1β production in a NLRP3- and ASC-dependent manner (72). Besides, in unprimed bone marrow-derived macrophages, *C. pneumonia* infection causes IL-1β maturation and production through NLRP3/ASC/caspase-1 pathway (73); however, *Orientia tsutsugamushi* triggers IL-1β production in macrophages merely *via* the activation of ASC inflammasome instead of NLRP3 (74). Overall, these mentioned findings indicate that different cell types and/or stimuli make multitudinous patterns of NLRP3 activation and IL-1β production. Therefore, it would be highly interesting to conduct comparative studies on the effects of betaine on canonical NLRP3 activation and IL-1β production of innate immune cells of different origins (e.g., monocytes, macrophages, and microglia) and/or under different conditions (e.g., unprimed/primed, infection, and injuries).

### Betaine in non-canonical inflammasome-mediated processing of IL-1β

Intriguingly, additional caspases and modulators (e.g., caspase-8/11) have emerging roles in inflammasome-mediated IL-1β maturation. Caspase-8, which regulates extrinsic apoptosis in response to TNF receptor 1 (TNFR1) and Fas activation (75), also modulates pro-IL-1β cleavage at exactly the same site as caspase-1 (76–78). In myeloid cells, Fas engagement triggers caspase-8-dependent IL-1β production through pathway that is fully independent of caspase-1 (79). Although caspase-8 and caspase-1 share the same cleavage site, the caspase-8-dependent IL-1β production does not require caspase-1 participation. CrmA (an inhibitor for caspase-8) inhibits the generation of IL-1β induced by LPS, though the exact mechanism still need to be unraveled (76). Furthermore, an *in vitro* study reported that both canonical and non-canonical (caspase-11 dependent) inflammasome activation and down-stream IL-1β processing are extremely restrained in *RIP3*^−/−^× *Caspase-8*^−/−^ cells (80). Interestingly, in endoplasmic reticulum (ER) stress, caspase-8-mediated IL-1β maturation does not need ASC expression (81).

**Figure 2:**
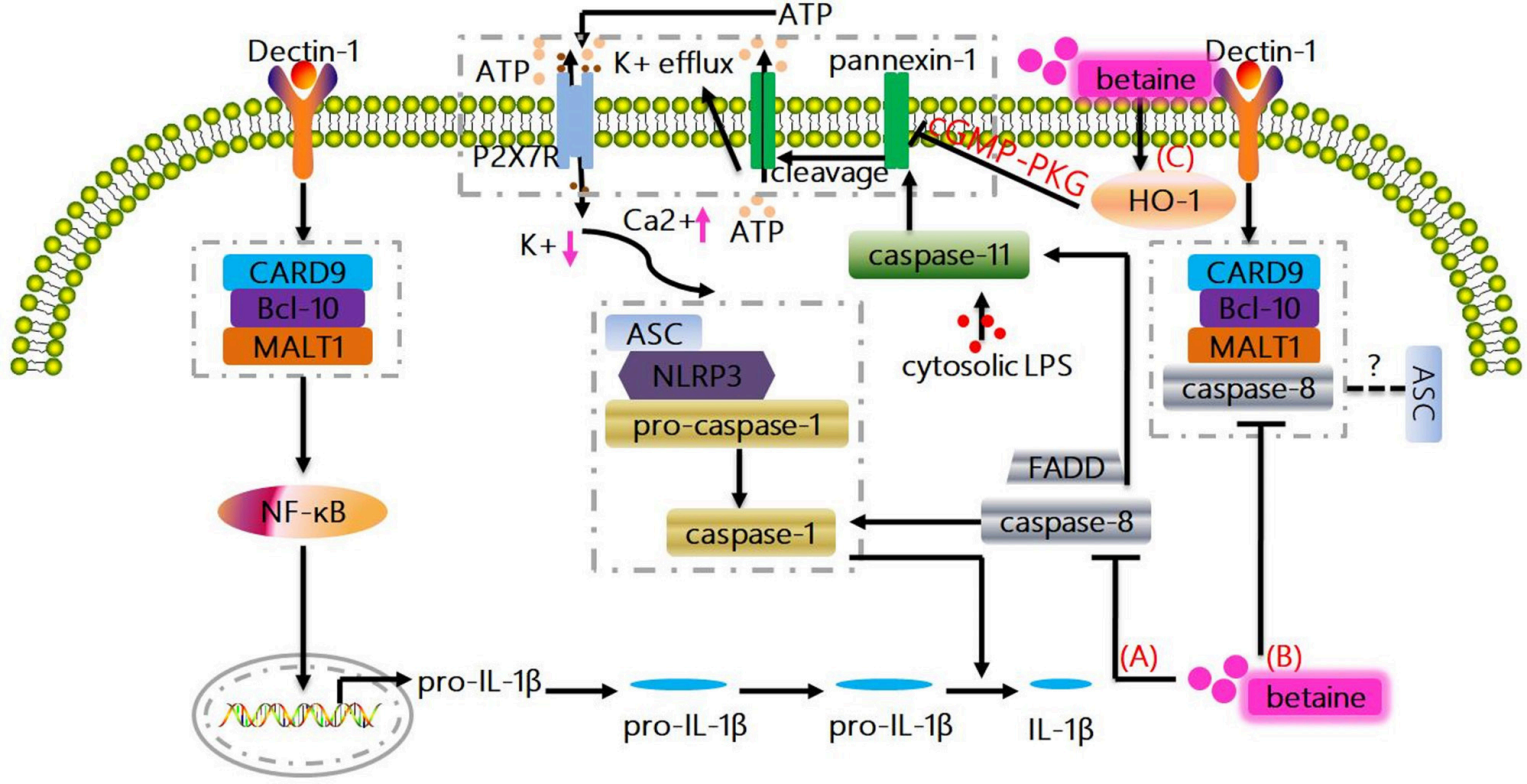
Non-canonical mechanisms whereby betaine inhibits IL-1β production. **(A)** Caspase-8 plays a vital role in promoting NF-κB signaling, and also modulates pro-IL-1β cleavage at exactly the same site as caspase-1. Actually, FADD and caspase-8 control the transcriptional priming of the NLRP3 inflammasome *via* modulation of the NLRP3 and pro-IL-1β expression. Betaine inhibits IL-1β production by preventing the induction of caspase-8 activity/activation. **(B)** The triggering of dectin-1 promotes Syk-dependent formation of the CARD9-Bcl-10-MALT1 scaffold that induces NF-κB activation and IL-1β transcription; as well as the formation and activation of a MALT1-caspase-8-ASC complex that mediates the processing of pro-IL-1β. Betaine reduces IL-1β production through suppressing the formation and activation of MALT1-caspase-8-ASC complex. **(C)** Caspase-11 is also essential for the NLRP3 inflammasome activation culminating with IL-1β production. Mechanistically, LPS-activated caspase-11 induces plasma membrane channel pannexin-1 cleavage, leading to K^+^ efflux and ATP release that interacts with P2X7R to promote the activation of NLRP3. Interestingly, betaine reduces IL-1β production by increasing HO-1 expression to activate cGMP-PKG signaling pathway, which ameliorates the pannexin-1 channel activity. HO-1, heme oxygenase-1; PKG, protein kinase G.

Under healthy conditions, caspase-8 is usually present in monomeric form as an inactive enzyme; however, the binding of Fas-associated death domain (FADD) to death receptors facilitates the recruitment of monomeric caspase-8 zymogens, which in turn causes caspase-8 homodimerization and subsequent caspase-8 activation (75). Except for exerting its classic function in apoptosis, caspase-8 also plays a vital role in promoting NF-κB signaling in antigen-stimulated T and B cells (75). Similarly, FADD and caspase-8 control the transcriptional priming of the NLRP3 inflammasome *via* modulation of the NLRP3 and pro-IL-1β expression. Multiple of reports have been demonstrated that betaine significantly blocks caspase-8 activation and/or reduces caspase-8 activity (59, 82, 83). Thus, betaine may inhibit IL-1β production by preventing the induction of caspase-8 activity/activation (Figure 2A). In fungi- and/or mycobacteria-stimulated dendritic cells (DCs), the triggering of dectin-1 can promote Syk-dependent formation of the CARD9-Bcl-10-MALT1 scaffold that induces NF-κB activation and IL-1β transcription; as well as the formation and activation of a MALT1-caspase-8-ASC complex that mediates the processing of pro-IL-1β (77, 84). As betaine blocks the activation of caspase-8, therefore, we suggest that betaine reduces IL-1β production through suppressing the formation and activation of MALT1-caspase-8-ASC complex (Figure 2B), though there are no current evidence on the effects of betaine on CARD9, Bcl-10, and MALT1, respectively. Summarily, considering caspase-8 controls human macrophage differentiation (85) and human monocyte and microglia activation (86, 87), it is obvious that caspase-8 participates in regulation of cytokines production by these immune cells. Indeed, caspase-8-deficient macrophages and/or DCs are hyperresponsive to TLR activation, and caspase-8 is required for normal M1 macrophage polarization whose markers include IL-1β [Ref. (88)]. Betaine appears to inhibit IL-1β production by reducing caspase-8 activity; however, no specific mechanisms (e.g., RIPK1/caspase-8/RIPK3/MLKL) by which betaine prevents the induction of caspase-8 activation have been found so far (85, 86).

Likewise, a study reported that the *caspase-1*^−/−^ mice is also deficient in caspase-11 (an executioner caspase which promotes pyroptosis or cell death; human orthologues caspase-4/5) expression (89–91). In *caspase-1*^−/−^ and *caspase-11*^−/−^ cells, it turned out that caspase-11 is required for caspase-1 activation and IL-1β maturation in response to exogenous stimulus, like *E. coli* and toxin (89, 92, 93). Mechanistically, in mouse macrophages and/or human myeloid cells, the direct interaction between pro-caspase-11 or pro-caspase-4/5 and LPS by binding of the CARD motif of pro-caspase-11 or pro-caspase-4/5 and the lipid A tail of LPS results in non-canonical inflammasome assembly (Figure not shown) (94–96). Moreover, a non-canonical pathway involving caspase-11 is also essential for the NLRP3 inflammasome activation culminating with IL-1β production. For instance, enterohemorrhagic *E. coli* (EHEC) infection induces the activation of caspase-11 in NLRP3 inflammasome *via* TRIF-dependent pathway in bone marrow-derived macrophages (93). In LPS-primed bone marrow-derived macrophages, LPS-activated caspase-11 (functions as a cytosolic LPS sensor) triggers plasma membrane channel pannexin-1 cleavage, resulting in K^+^ efflux and ATP release that interacts with P2X7R to promote the activation of NLRP3. Notablly, the caspase-11/pannexin-1/NLRP3 is considered as an important mechanism for IL-1β production (97). Indeed, pannexin-1 channel activity can be attenuated by NO and heme oxygenase-1 (HO-1) *via* activating cGMP-protein kinase G (PKG) signaling pathway (98–100). Interestingly, betaine directly increases the expression levels of HO-1, and this effect may inhibit the NLRP3 inflammasome (101). Taken together, betaine may reduce IL-1β production by increasing HO-1 expression to activate cGMP-PKG signaling pathway, which ameliorates the pannexin-1 channel activity (Figure 2C). Obviously, this potential mechanism needs to be completely elucidated. The delivered intracellular LPS could significantly trigger caspase-11 non-canonical inflammasome activation and IL-1β production in a type I IFN signaling-independent manner (102). Thus, it is interesting to investigate whether betaine can influence gene expression induced by type I IFNs which is responsible for cytoplasmic sensing of LPS by caspase-11 in the future.

### Betaine in inflammasome-independent sources of IL-1β

As described in aforementioned sections, inflammasome formation associated with caspase-1 and/or caspase-11 is the most important mechanism for the processing of IL-1β. However, inflammasome-independent ways also affect inflammation and diseases from the observations (uncompleted abrogation of IL-1β production) found in deletion of caspase-1/11 in inflammatory disease models (e.g., osteomyelitis and arthtitis) (103–105). Cathepsin C/G, elastase, chymase, and proteinase-3 are all responsible for cleaving pro-IL-1β into activated IL-1β (106, 107). Mechanistically, in previous mentioned inflammatory diseases, cathepsin C uniquely modulates inflammasome-independent IL-1β production and genetic deletion of cathepsin C significantly lowers IL-1β levels (108). Pharmacological inhibition of elastase and chymase diminish IL-1β production (105). Also, genetic and pharmacological inhibition of proteinase-3 has critical role in mitigating IL-1β-mediated inflammation (107). However, no lines of evidence (direct or indirect) present the effects of betaine in inflammasome-independent sources of IL-1β currently.

## Betaine inhibits IL-1β release

Release of mature IL-1β into the extracellular environment is essential for IL-1β to exert its host defense function in response to infections and injuries. The mature IL-1β release depends on the non-canonical pathways of export from the cytosol (109–111). As lack of conventional signal peptide, IL-1β cannot target to the conventional ER-Golgi secretory pathway as the same as other cytokines resulting in the accumulation of IL-1β in cytosol (109). In this section, we summarize the influences of betaine in four main possible mechanisms for IL-1β release, including exocytosis *via* secretory lysosomes, microvesicle shedding from plasma membrane, release of exosomes, and passive efflux across leaky plasma membrane during pyroptotic cell death.

### Betaine in exocytosis of IL-1β-containing secretory lysosomes

**Figure 3:**
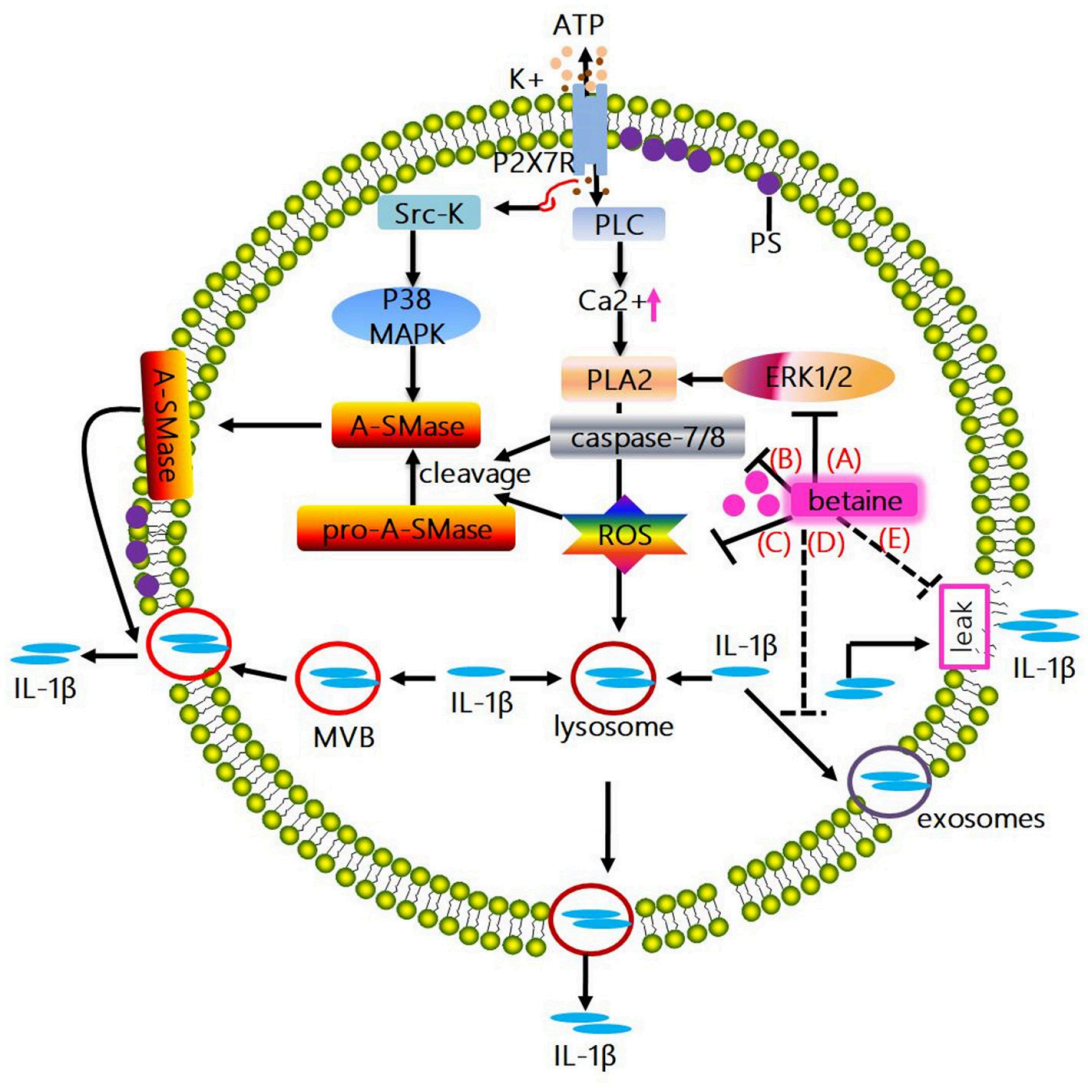
Mechanisms whereby betaine inhibits IL-1β release. **(A)** The activation of ATP-mediated P2X7R promotes IL-1β and caspase-1 localize to secretory lysosomes together with the lysosomal enzymes secretion. Mechanistically, the above process is associated with the P2X7R-induced K^+^ efflux, which enhances the activation of PLC, thus increasing intracellular Ca2^+^ concentration, enabling Ca2^+^-dependent PLA2 activation and promoting exocytosis of the IL-1β-containing lysosomes. Actually, PLA2 activity has been showed to be regulated by phosphorylation by ERK1/2 and ROS. Betaine inhibits IL-1β release *via* suppressing exocytosis of the IL-1β-containing lysosomes through reducing the activity of PLA_2_ by blocking the ERK1/2 signaling pathway and/or lowering the ROS level. **(B)** ATP-induced P2X7R activation promotes the C-terminal domain interacts with Src-K, phosphorylating the subsequent p38 MAPK, inducing acidic A-SMase delivers from the inner to the outer plasma membrane. Subsequently, A-SMase hydrolyzes sphingomyelin to generate ceramide, altering membrane fluidity, promoting the formation of plasma membrane blebs and resulting in shedding of IL-1β-containing microvesicles. And microvesicle shedding is preceded by flip of PS to the outer leaflet of the plasma membrane. Interestingly, A-SMase can be activated by proteolytic cleavage of pro-A-SMase by caspase-8 and caspase-7 or ROS. Betaine inhibits IL-1β release *via* blunting the IL-1β-containing microvesicle shedding by blocking the activation of A-SMase through inhibiting caspase-7/8 activation. **(C)** Betaine inhibits IL-1β release by restraining the IL-1β-containing microvesicle shedding by reducing ROS level. **(D)** IL-1β release is also involving exocytosis of exosomes; besides, MVBs formation and IL-1β and caspase-1 accumulation can be tightly modulated by inflammasome complex. Betaine lowers the release of IL-1β by inhibiting the formation of MVBs and exosomes *via* inhibiting the NLRP3 inflammasome activation. **(E)** Moreover, IL-1β is passively released alongside DAMPs following plasma membrane rupture. Betaine blunts the passive efflux of IL-1β through its effects on protecting cell membrane from external membrane-perturbing compounds-induced rupture. PLA_2_, phospholipase A_2_; ERK1/2, extracelluar signal-regulated kinase; A-SMase, acidic sphingomyelinase; ROS, reactive oxygen species; MVBs, multivesicular bodies.

A study conducted in 1990 presented the evidence that in activated human monocytes, inhibition of protein transport and secretion through the ER-Golgi formation of endo-membrane system has little effect on IL-1β release (109); however, the IL-1β release is closely related to secretory lysosomes (109). Indeed, the exocytic process can be stimulated by ATP (released from dying cells, etc.), and the subsequent migration of exocytic lysosomes to the plasma membrane allow the content (e.g., IL-1β) trapped in lysosomes, to secret into extracellular compartment (112). Moreover, in human monocytes and mouse macrophages, once ATP-mediated P2X7R activation, the IL-1β and caspase-1 localize to secretory lysosomes and secrete with the lysosomal enzymes (113, 114). Mechanistically, the above process is related to the P2X7R-induced K^+^ efflux, which leads to the activation of phosphatidylcholine-specific phospholipase C (PLC) to increase intracellular Ca^2+^ concentration, resulting in Ca^2+^-dependent phospholipase A_2_ (PLA_2_) activation and exocytosis of the IL-1β-containing lysosomes (115). The aforementioned processes can be blocked by using the inhibitors of phosphatidylcholine-specific PLC and/or PLA_2_. Indeed, in mouse macrophages and/or rat astrocytes, PLA_2_ activity has been showed to be regulated by phosphorylation by ERK1/2 and ROS (116, 117). Based on previous discussed section, betaine reduces ROS level in stressed cells; and the ERK1/2 signaling pathway could be shut off by betaine in adipogenic-differentiated C2C12 cells (118). Thus, we speculate that betaine may slow down IL-1β release *via* suppressing exocytosis of the IL-1β-containing lysosomes through reducing the activity of PLA_2_ by blocking the ERK1/2 signaling pathway (Figure 3A) and/or lowering the ROS level (Figure 3C). However, this needs experimental validation, and the exact mechanisms by which betaine regulates the IL-1β release through inhibiting lysosome exocytosis remain to be revealed.

### Betaine in shedding of IL-1β-containing plasma membrane microvesicles

The *in vitro* cell models (e.g., THP-1 monocyte, DCs, and microglia) stimulated by P2X7R have demonstrated that a mechanism for IL-1β release depends on shedding of plasma membrane microvesicles (119–121). Mechanistically, ATP-induced P2X7R activation enhances the C-terminal domain interaction with src-protein tyrosine kinase (Src-K) to phosphorylate the subsequent p38 MAP kinase (p38 MAPK), inducing acidic sphingomyelinase (A-SMase) delivery from the inner to the outer plasma membrane. Subsequently, A-SMase hydrolyzes sphingomyelin to generate ceramide, altering membrane fluidity, promoting the formation of plasma membrane blebs and resulting in shedding of IL-1β-containing microvesicles (122). Shed microvesicles possess many phospholipids and proteins [e.g., phosphatidylserine (PS), P2X7R, pro-caspase-1, pro-IL-1β, and IL-1β]. Microvesicle shedding is preceded by flip of PS to the outer leaflet of the plasma membrane; however, the exact mechanism by which IL-1β effluxes out of the microvesicles is still obscure. Interestingly, betaine takes part in the above mentioned processes. A-SMase can be activated by proteolytic cleavage of pro-A-SMase by caspase-8 and caspase-7 (123) or ROS (124, 125). Based on the above discussed section, betaine significantly blocks caspase-8 activation and/or reduces caspase-8 activity (59, 82, 83); and attenuates caspase-7 activation (126), and lowers ROS level. Therefore, betaine seems to inhibit IL-1β release *via* blunting the IL-1β-containing microvesicle shedding by blocking the activation of A-SMase through inhibiting caspase-7/8 activation (Figure 3B) and/or reducing ROS level (Figure 3C). However, these findings are mainly found in non-immune cells (e.g., PC12 cells) and the possible mechanisms whereby betaine targets microvesicles shed from innate immune cells are not currently available.

### Betaine in exocytosis of IL-1β-containing exosomes

Exosomes is the fusion of multivesicular bodies (MVBs) with the cell plasma membrane. A non-canonical pathway for IL-1β release involving exocytosis of exosomes is also found in P2X7R-stimulated macrophages, DCs and B-lymphocytes (127); and pro-IL-1β, pro-caspase-1, bioactive caspase-1, IL-1β, MHCI, and MHCII [a feature of exosomes originated from antigen presenting cells (APCs)] do exist in the exosomes secreted from these cells (127). Interestingly, a study reported that the release of IL-1β and MHCII can be significantly blocked in ASC^−/−^ and NLRP3^−/−^ mice (128). Thus, it seems that MVBs formation and IL-1β and caspase-1 accumulation can be tightly modulated by inflammasome complex. Notably, the aforementioned section indicate that betaine inhibits NLRP3 inflammasome activation, thus we suggest that betaine may lower the release of IL-1β by inhibiting the formation of MVBs and exosomes, though the specific mechanisms still remain to be identified (Figure 3D).

### Betaine in passive efflux of IL-1β across hyperpermeable plasma membrane during pyroptotic cell death

IL-1β release is closely related to a loss in membrane integrity during pyroptotic cell death (129–132). Due to the caspase-1/11 drives cell apoptosis and/or pyroptosis and IL-1β cleavage, IL-1β is passively released alongside DAMPs following plasma membrane rupture (133–135). Indeed, ATP-mediated IL-1β release but not its processing can be absolutely blocked by punicalagin which functions as an inhibitor to limit plasma membrane damage induced by external membrane-perturbing compounds (132). Likewise, betaine is essential for maintaining cell membrane integrity and serves as an osmolyte that regulates cell volume and protects cells from environmental stresses (10, 12), and inhibits various hyperosmotic-induced apoptosis-related proteins (e.g., caspase-3/8/9) activity in MDCK cells (83). Therefore, betaine may blunt the passive efflux of IL-1β through its effects on protecting cell membrane from external membrane-perturbing compounds-induced rupture, though the exact mechanism is still not clear (Figure 3E).

## Concluding remarks

**Figure 4:**
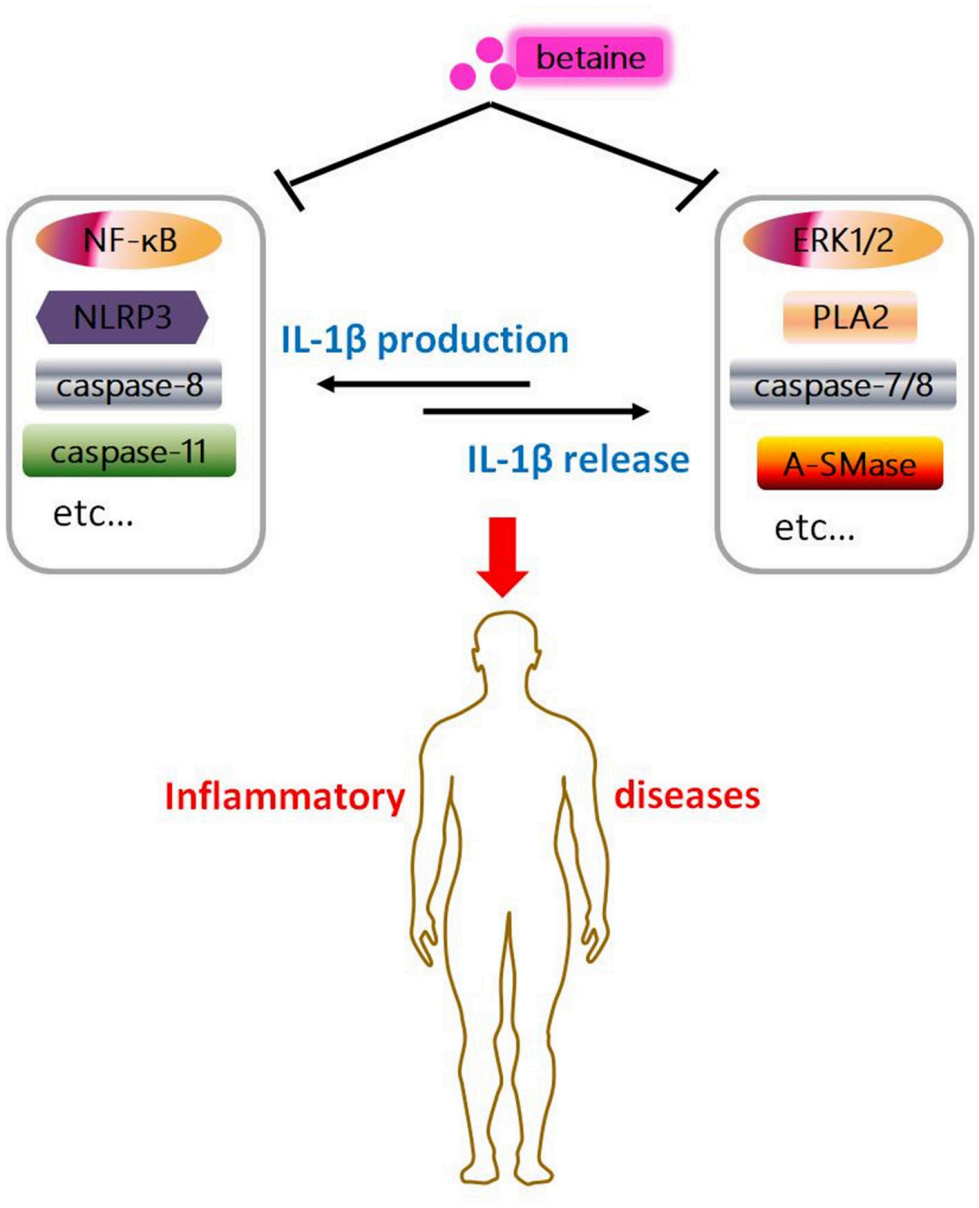
Graphical abstract of betaine in IL-1β secretion. Betaine inhibits IL-1β production and release through various pathways, respectively. And it indicates betaine functions as a dietary adjuvant therapy in diverse inflammatory diseases involving IL-1β secretion.

IL-1β plays overarching roles in stimulation of innate immune system and inflammatory processes/diseases (136, 137). Various nutrients have proven to be effective in modulation of inflammation and inflammatory diseases by lowering IL-1β secretion (138). Betaine is a stable and nontoxic natural nutrient and has anti-inflammatory effects (16). Mechanistically, betaine inhibits IL-1β production through various pathways, such as NF-κB, canonical NLRP3, and caspase-8/11 (Figures 1, 2). Betaine also inhibits IL-1β release *via* pathways including ERK1/2/PLA_2_, caspase-8/A-SMase, MVBs and exosomes (Figure 3). Therefore, it is meaningful to develop betaine as a dietary adjuvant therapy in diverse inflammatory diseases involving in IL-1β secretion (Figure 4). Inflammasome-independent pathway also affects inflammatory process and inflammatory diseases; thus, it is worthy of investigating the effects of betaine in inflammasome-independent sources of IL-1β. Additionally, the P2X7R is responsible for ATP-mediated mature IL-1β release (139); however, whether betaine affects IL-1β release by influencing P2X7R activity remains to be revealed. A study showed that caspase-11 controls IL-1β release through degradation of transient receptor potential channel (TRPC) 1 (140); nevertheless, no current relation between betaine and TRPC1 has been found. Given betaine alters gene expression/function *via* epigenetic modifications [e.g., miRNAs and DNA methylation (141)], therefore, it is interesting to further study the involvement of epigenetic modification in the effects of betaine in inhibiting IL-1β production and release.

## Author contributions

YX and WR designed the review article, and YX wrote the review article. RH, YY, and WR revised the review article. YX, SC, and GZ helped with designing figures and finding relevant literature. WR approved the final manuscript.

### Conflict of interest statement

The authors declare that the research was conducted in the absence of any commercial or financial relationships that could be construed as a potential conflict of interest.

## Abbreviations

IL: interleukin
BHMT: betaine-homocysteine methyl transferase
NF-κB: nuclear factor kappa B
NLR: NOD-like receptor
TLRs: toll-like receptors
TNF: tumor necrosis factor
MAPKs: mitogen-activated protein kinases
NIK/IKK: nuclear factor-including kinase/IκB kinase
JNK: c-Jun NH2-terminal kinase
p38: protein 38
ERK: extracelluar signal-regulated kinase
HMGB1: high-mobility group box 1
HDAC3: histone deacetylases 3
AIM2: absent in melanoma 2
ALR: AIM2-like receptor
PAMP: pathogen-associated molecular pattern
DAMP: danger-associated molecular pattern
P2X7R: purinergic ligand-gated ion channel 7 receptor
FOXO1: forkhead box O 1
TXNIP: thioredoxin interacting protein
ROS: reactive oxygen species
IRS-1: insulin receptor substrate 1
ER: endoplasmic reticulum
FADD: Fas-associated death domain
PKG: protein kinase G
HO-1: heme oxygenase-1
PLC: phospholipase C
PLA_2_: phospholipase A_2_
Src-K: src-protein tyrosine kinase
A-SMase: acidic sphingomyelinase
PS: phosphatidylserine
MVBs: multivesicular bodies
APCs: antigen presenting cells.
